# The interrelationship between water access, exclusive breastfeeding and diarrhea in children: a cross-sectional assessment across 19 African countries

**DOI:** 10.7189/jogh-11-04001

**Published:** 2021-03-27

**Authors:** Paschal A Apanga, Ann M Weber, Lyndsey A Darrow, Mark S Riddle, Wei-Chen Tung, Yan Liu, Joshua V Garn

**Affiliations:** 1 School of Community Health Sciences, University of Nevada, Reno, Nevada, USA; 2 School of Medicine, University of Nevada, Reno, Nevada, USA; 3 The Valley Foundation of School of Nursing, San Jose State University, San Jose, California, USA

## Abstract

**Background:**

Access to an improved water supply and practicing exclusive breastfeeding are essential for improving maternal and child health outcomes. However, few studies have been equipped to assess the interdependencies between access to improved water, practicing exclusive breastfeeding, and child health. The primary aim of our study was to assess whether access to an improved water supply and water-fetching were associated with mothers’ practice of exclusive breastfeeding.

**Methods:**

We analyzed data on 247 090 mothers with children 5 months old or less using Multiple Indicator Cluster Surveys from 19 African countries. Multivariable logistic regression was used to estimate the relationship between our exposures and exclusive breastfeeding practice, while meta-analytic methods were used to pool adjusted estimates across 19 countries.

**Results:**

The prevalence of exclusive breastfeeding ranged from 22% in Nigeria to 70% in Malawi. Pooled results showed water-fetching was not associated with exclusive breastfeeding (adjusted prevalence odds ratios (aPOR) = 1.04, 95% confidence interval (CI) = 0.89, 1.21). Access to an improved water source was also not associated with exclusive breastfeeding (aPOR = 1.06, 95% CI = 0.94, 1.21). Across all countries many women were spending a significant amount of time water-fetching each day (mean time varied from 20 minutes in Ghana to 115 minutes in Mauritania). Exclusively breastfed children had 33% lower odds of diarrhea than those who were not exclusively breastfed (aPOR = 0.67, 95% CI = 0.56, 0.78).

**Conclusion:**

Our study is the first to assess the relationship between access to improved water supply, water-fetching and exclusive breastfeeding. We found that access to water supply and time spent by mothers fetching water were not associated with exclusive breastfeeding practice, even though mothers spent significant time fetching water.

Ingestion is a particularly important pathway for acquisition of enteropathogens that cause acute diarrheal illness, which is responsible for ~ 10%-12% of all deaths in children under 5 years of age [1,2]. Interventions that provide microbiologically clean water can act as a barrier to pathogen acquisition [3]. Exclusive breastfeeding (EBF) may also act as a barrier, and simultaneously builds children’s immunity, protecting them from diarrheal and respiratory diseases and improving their response to vaccination [4-7]. Both access to microbiologically safe water and practicing exclusive breast feeding are important, but few studies have been equipped to assess the interdependencies between these.

It is not known whether household water-fetching is associated with a woman’s practice of EBF or time available to exclusively breastfeed. Women without adequate water spend much of their time and energies water-fetching [8,9], which could compete with time to exclusively breastfeed. There is some evidence exploring the links between water-fetching and other health practices, such as antenatal care attendance, and health facility delivery [10]. There are few studies assessing the effects of water-fetching on diarrhea in children [10-12], while most studies focus on the high risk of contamination [13-15]. However, it’s not known how time spent water-fetching affects the practice of EBF.

There are a number of perceptions about water source types and water quality that might lead to women introducing water prematurely. It has been reported that many women believe that infants can be given water if the water is clean [16]. Similarly, women who perceive that their own water quality is good are more likely to drink that water compared to women believing that their water quality is poor [17,18]. Further, perceptions of water cleanliness might not align with if the water is actually microbiologically safe. It is widely perceived that piped water is the “ideal” compared to other water sources, although contaminated piped water has been reported in a range of settings [19]. Similarly, women who have access to improved water sources might be better protected from contaminants compared to their counterparts with unimproved water sources, but not all improved water sources are safe or consistently safe [19,20].

Even when an infant is exclusively breastfed, they may be at increased risk of infection if other household members are exposed to microbiologically contaminated water. Lack of safe water, sanitation, and hygiene (WASH) accounts for an estimated 88% of diarrhea-associated mortality in young children [21,22], and many of these deaths are children who were exclusively breastfed, but living in families with high prevalence of WASH preventable diseases. Children within the first two years of life are most vulnerable, with a decline in mortality as the child grows older [23]. Interventions that provide water supply improvements are therefore essential in reducing diarrheal diseases among young children [24].

This study characterizes the pathways between improved water access, water-fetching, EBF, and diarrhea in children living in 19 African countries, using the Multiple Indicator Cluster Surveys (MICSs) [25]. Our primary aims were to assess the association between access to improved water supply and EBF practice, and between water-fetching and EBF during the first five months after birth. Our secondary aims were to characterize the association between access to an improved water supply and diarrhea prevalence, and between EBF and diarrhea prevalence in children five months old or less.

## METHODS

### Study design, setting and data collection

We used data from the MICS from 19 African countries, collected between 2013 and 2019. The MICS is a nationally representative household cross-sectional study conducted in many countries around the world, with systematically collected data on women and children [25]. Our study population was mothers with children five months old or less living in the following African countries: Democratic Republic of Congo (DRC), Gambia, Ghana, Lesotho, Madagascar, Sierra Leone, Togo, Zimbabwe, Cameroon, Congo, Côte d'Ivoire, Benin, Guinea Bissau, Guinea, Malawi, Mali, Mauritania, Nigeria, and Sudan. Most of the countries (12) were from West Africa, three from East Africa, one from North Africa, one from Southern Africa, and two from Central Africa.

The MICS employs a two-stage sampling technique in each country. In the first stage, census enumeration areas were selected from each sampling strata using probability proportional to size of the number of households in each enumeration area. The second stage of sampling involved selection of households using systematic random sampling from each enumeration area, forming survey clusters. Household participation rates are usually 90%-95% [25]. Detailed description of the MICS sampling design and procedures are published elsewhere [26,27].

### Outcome measures

Our primary outcome of interest was exclusive breastfeeding practice. Exclusive breastfeeding was dichotomized as “yes” for infants five months of age or less who were still being breastfed and did not receive other fluids/foods in the past 24 hours, and categorized as “no” for infants five months of age or less who were not being breastfed, or were still being breastfed but received other fluids/foods in the past 24 hours [28]. Our secondary outcome was caregiver-reported diarrhea in the past two weeks. This variable was also dichotomized as “yes” for infants whose parents had reported diarrhea in the past two weeks and “no” for infants who did not have diarrhea.

### Predictors

The predictors in our study included time spent in water-fetching, any water-fetching, and having an improved water source. “Water-fetching” in our study means spending some time outside the household premises to go get water and return to the household. Round-trip time spent by mothers fetching water was categorized into two levels as: round-trip time greater than 30 minutes and round-trip of 30 minutes or less as the referent category; 30 minutes was chosen as the cutoff time to align with the basic drinking water definition used by the World Health Organization (WHO)/ United Nations Children's Fund (UNICEF) Joint Monitoring Programme for Water Supply, Sanitation and Hygiene (JMP) [29]. We also compared any water-fetching, to mothers who did not water-fetch (ie, either the water was close and did not require fetching or someone else in the household fetched the water). We used the definition of improved water, as defined by the JMP, where improved sources include piped water, boreholes or tube wells, protected dug wells, protected springs, tanker-truck, rain water and packaged water, whereas unimproved water sources include unprotected dug wells, springs, and surface water collected directly from river, dam, lake, pond, stream, canal and irrigation channels [29].

### Covariates

Covariates of interest in our data included: educational level of mothers, age of the mother, household wealth, sanitation and residential status. Mother’s educational level was categorized as no education, primary, secondary and college or higher education. Household wealth was expressed in wealth quintiles as a composite indicator of wealth derived from principal component analysis using household assets [26]. We categorized wealth quintiles as upper two, middle and lower two wealth quintiles. Sanitation was also categorized as improved vs unimproved sanitation according JMP’s definition of basic sanitation [30]. Residential status was recorded as either urban or rural.

### Data analysis

Characteristics of study participants across 19 African countries were presented as counts and percentages (if categorical) and as mean and standard deviation (if continuous). Multivariable logistic regression was used to assess the relationships between the variables of interest and outcome variables, while controlling for potential confounders. In estimating the relationship between access to improved water sources, and outcomes (EBF and diarrhea), and between household water-fetching and EBF, we controlled for educational level of the mothers, household wealth, sanitation and residential status. When assessing the relationship between EBF and diarrhea prevalence in children five months old or less, we adjusted for the educational level of the mothers, household wealth, sanitation, maternal age, child’s age and residential status. We specified all our potential confounders *a priori,* as we thought there was biological plausibility that they might be associated with both the exposure and outcome of interest. We used survey procedures in SAS to account for the stratified design (ie, strata, clusters, and sampling weights) for all analyses.

We conducted a sensitivity analysis on the association between round-trip time spent by mothers fetching water and EBF to see whether there was a dose response relationship with increasing time spent. We compared EBF prevalence among mothers who spent a round-trip of between 30 minutes and 60 minutes, and a round trip of greater than 60 minutes, to mothers who spent a round-trip of 30 minutes or less as the referent category [29].

We also tested whether child (age categories 0-1, 2-3 and 4-5 months of age) [4], moderates the role of time spent by mothers fetching water on EBF. As a secondary descriptive analysis, we also show the prevalence of EBF among mothers with children of different age categories (0-1, 2-3, 4-5 &≤5 months), and the mean time spent by mothers during water-fetching. Data were analyzed using SAS version 9.3 (SAS Institute, Cary, NC) for descriptive statistics and multivariable logistic regression.

### Synthesis of Results across all countries

Random-effects meta-analysis with inverse variance weighting was used to pool adjusted odds ratios estimates of our key relationships across all 19 countries. We reported statistical heterogeneity using the I^2^ statistics. An I^2^>50% may be of substantial heterogeneity, while that of an I^2^>75% may be of considerable heterogeneity [31]. We present results both by each country and overall using forest plots. The meta-analysis was conducted using Stata 16 SE (Stata Corp, College Station, TX, USA).

## RESULTS

### Descriptive statistics

The MICS data from the 19 countries was restricted to a total 247 090 mothers with children 5 months old or less living in a mix of rural and urban areas (**Table 1**). The mean age of mothers across the countries ranged from 25-29 years. Twelve of the countries (Gambia, Ghana, Lesotho, Sierra Leone, Togo, Côte d'Ivoire, Guinea Bissau, Guinea, Malawi, Mauritania, Nigeria, and Sudan) had majority of mothers with no formal education (**Table 1**). The prevalence of EBF ranged from 22% in Nigeria to 70% in Malawi and EBF prevalence was at least 50% in 9 of the 19 countries (**Table 1**). Not surprisingly, the prevalence of EBF decreased with increasing age of the child across all countries (Figure S1 in the **Online Supplementary Document**). The prevalence of any breastfeeding (ie, currently breastfeeding, without regard to supplemental food or water) among mothers with children 5 months old or less ranged from 97% to 100% across countries (**Table 1****).** The mean time spent by mothers fetching water ranged from 20 minutes in Ghana to 115 minutes in Mauritania (**Table 1**).

**Table 1:** Characteristics of mothers with children five months old or less across 19 countries (n = 247 090).

Country	Benin	Cameroon	Congo	Côte d'Ivoire	DRC	Gambia	Ghana	Guinea	Guinea Bissau	Lesotho	Madagascar	Malawi	Mali	Mauritania	Nigeria	SierraLeone	Sudan	Togo	Zimbabwe
Survey year	2014	2014	2014-2015	2016	2017-2018	2018	2017-2018	2016	2014	2018	2018	2013-2014	2015	2015	2016-2017	2017	2014	2017	2019
Sample size	10 191	8742	8719	8961	17 741	8306	12 146	8287	6556	7427	17 933	24 464	14 745	11 724	30 749	13 727	20 013	7456	9203
Age; mean (SD*)	29 (10.8)	27 (9.6)	29 (10.2)	28 (9.6)	27 (9.6)	25 (8.9)	27 (10.5)	28 (9.8)	26 (9.5)	27 (10.2)	27 (10.1)	28 (10.1)	26 (9.0)	27 (10.2)	28 (10.0)	27 (9.8)	27 (9.7)	28 (10.4)	28 (10.4)
EBF Prevalence; (%)	39	28	34	22	55	54	43	34	53	57	51	70	33	40	22	50	57	67	42
Currently breastfeeding; (%)	99	99	100	99	99	99	98	100	99	90	99	99	99	97	98	99	99	100	99
Mean time water-fetching* (SD)	34 (5.2)	28 (2.4)	30 (1.4)	31 (1.6)	39 (1.8)	23 (2.0)	20 (1.8)	52 (3.0)	53 (6.8)	33 (2.4)	24 (1.4)	56 (2.3)	41 (4.6)	115 (11.0)	45 (4.2)	40 (3.9)	87 (7.2)	23 (1.4)	35 (1.3)
**Education; (%)**																			
None	65	28	13	68	25	64	42	80	62	44	28	24	81	68	46	70	57	45	7
Primary	20	34	31	19	37	14	18	9	24	22	50	62	9	22	21	10	26	34	40
Secondary or higher	15	38	56	14	38	21	40	11	14	34	22	14	10	11	32	21	16	21	53
Missing		5	4	5	250	48	24	2	24	177	345	32	39	41	87	28	28	37	147
**Household’s wealth; (%)**																			
Lowest two quintiles	33	36	67	48	54	52	46	45	52	53	48	39	44	42	39	46	49	45	41
Middle quintile	17	22	14	22	21	16	19	21	20	18	20	21	18	23	20	22	21	21	21
Highest two quintiles	50	42	20	29	25	31	35	34	29	29	32	40	38	35	41	31	30	34	38
**Sanitation; (%)**																			
Unimproved sanitation	57	45	66	57	70	43	41	52	82	34	58	39	57	52	51	55	66	58	32
Improved sanitation	43	55	34	43	30	57	59	48	18	66	42	61	43	48	49	45	34	42	68
missing			4					3		1	2	13		38		6	38		
**Area of residence; (%)**																			
Rural	44	49	66	66	68	53	56	68	67	75	74	87	75	56	70	63	72	68	70
Urban	56	51	34	34	32	47	44	32	33	25	26	13	25	44	30	37	28	32	30

SD – standard deviation

*Mean time is time spent (in minutes) by mothers fetching water.

### Factors associated with prevalence of EBF and prevalence of diarrhea

**Figure 1:**
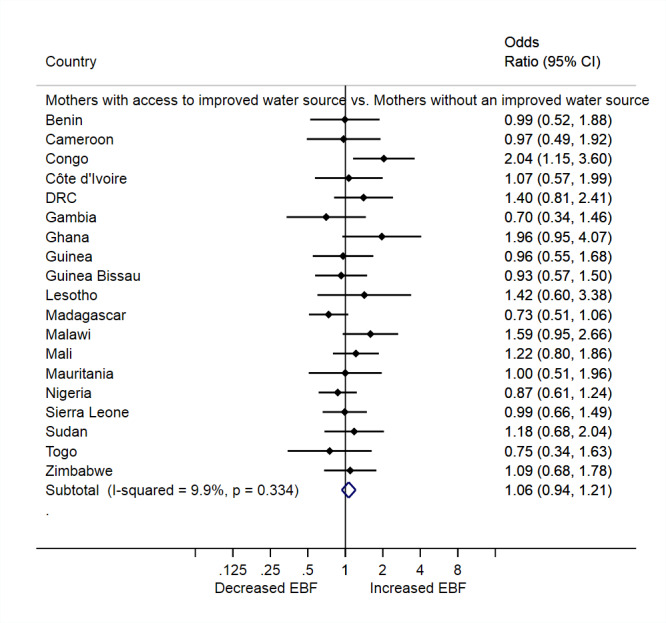
The association between access to an improved water source and exclusive breastfeeding (EBF) practice among mothers in 19 African countries.

The overall prevalence of EBF was similar between mothers with access to an improved water source and mothers without such access (adjusted prevalence odds ratios (aPOR):1.06, 95% CI = 0.94, 1.21), and there was little heterogeneity in the estimates across the 19 countries (I^2^ = 9.9%, *P* = 0.334). Most individual countries had null results when assessing the association between access to an improved water source and EBF, although mothers in Congo with access to an improved water source were twice as likely to exclusively breastfeed as compared to their peers with an unimproved water source (aPOR = 2.04, 95% CI = 1.15, 3.60; **Figure 1**).

**Figure 2:**
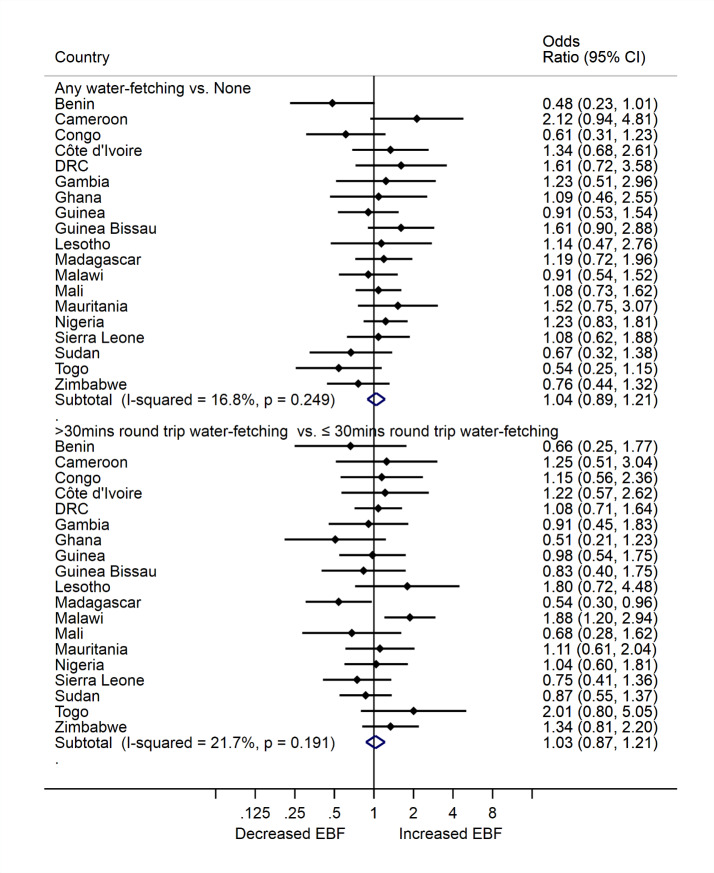
The association between any water-fetching and times spent by mothers fetching water and exclusive breastfeeding (EBF) practice among mothers in 19 African countries.

There was no relationship between water-fetching and EBF, regardless of how water fetching was categorized (**Figure 2**). The prevalence of EBF was similar comparing mothers who participated in any water-fetching to mothers who did not fetch water at all (aPOR = 1.04, 95% CI = 0.89, 1.21). All 19 countries had null findings, with low heterogeneity across estimates (I^2^ = 16.8%, *P* = 0.249). Similarly, the prevalence of EBF was comparable between mothers who spent greater than 30 minutes round-trip water-fetching and mothers who spent ≤30 minutes round-trip water-fetching (aPOR = 1.03, 95% CI = 0.87, 1.21), again with little heterogeneity in the estimates (I^2^ = 21.7%, *P* = 0.191; **Figure 2**). In Madagascar, mothers who spent greater than 30 minutes water-fetching had 46% lower odds of EBF compared to mothers who spent 30 minutes or less (aPOR = 0.54, 95% CI = 0.30, 0.96). In contrast, mothers in Malawi who spent greater than 30 minutes water-fetching had 88% higher odds of EBF compared to mothers who spent 30 minutes or less. Our sensitivity analysis to assess if there was a dose response between water-fetching time and EBF showed that prevalence of EBF was similar between various times spent water-fetching (Figure S2 in the **Online Supplementary Document**). Also, our sensitivity analysis to assess whether child age moderates the relationship between time spent by mothers fetching water and EBF showed similar prevalence of EBF among child age ranges 0-1, 2-3 and 4-5 months (Figure S3 in the **Online Supplementary Document**).

**Figure 3:**
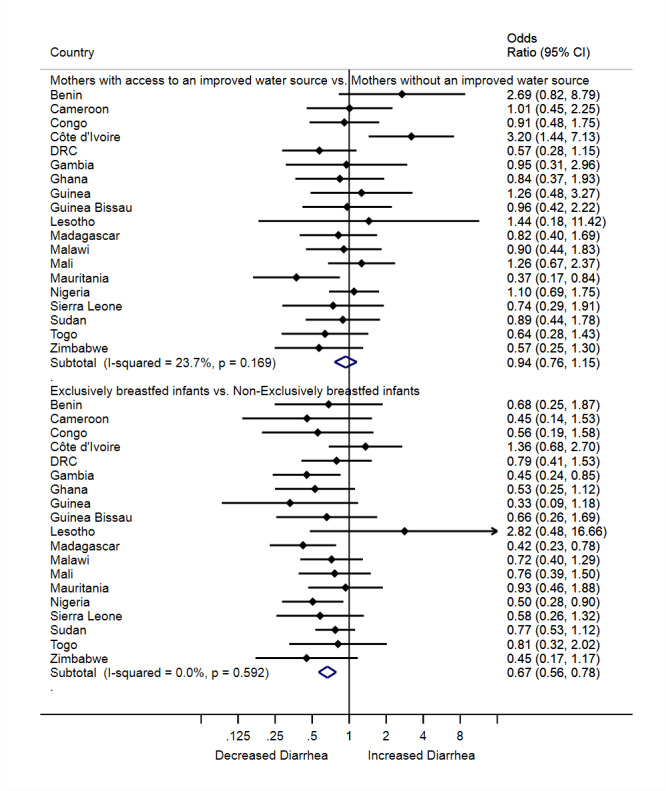
The association between access to improved water sources, exclusive breastfeeding practice, and diarrhea prevalence in children five months old or less in 19 African countries.

Overall, children who were exclusively breastfed had 33% lower odds of diarrhea than those not breastfed (aPOR = 0.67, 95% CI = 0.56, 0.78), and this finding was consistent across many of the countries (I^2^ = 0.00%, *P* = 0.592; **Figure 3****).** The prevalence of diarrhea in children five months old or less was similar between mothers with access to an improved water source and mothers without access to an improved water source (aPOR = 0.94, 95% CI = 0.76, 1.15; I^2^ = 23.7%, *P* = 0.169; **Figure 3**).

## DISCUSSION

The study assessed 247 090 mothers with children 5 months old or less from 19 countries in Africa, and offers new insight into whether water access and household water-fetching play a role in a mothers’ practice of EBF. Existing literature shows water-fetching leads to a significant burden on women in a number of areas [32], however, we did not observe water access or water-fetching to be associated with mothers’ practice of exclusive breastfeeding. The literature also describes the importance of breastfeeding on health [33] and our study is consistent in finding lower prevalence of diarrhea with those who practiced EBF. Across the 19 countries, time spent fetching water by mothers was often high, and the prevalence of EBF practice among mothers was often low.

We observed that only 9 out of the 19 countries met the WHO’s Global Nutrition Target of 50% EBF prevalence by 2025 [34]. The low prevalence of EBF in some of the countries in our study may be due to socio-cultural beliefs and misinformation about EBF. Barriers to EBF vary across communities, and stem from a variety of factors, including beliefs about breast milk being insufficient, beliefs about water quenching thirst, traditions of giving water as a welcoming, and other taboos and social norms [35-38]. Countries with low EBF prevalence in our study could benefit from increased promotion of EBF, education and support [39].

Our study also found that many mothers spent a significant amount of time water-fetching. Mothers on average spent more than 30 minutes round-trip water-fetching in 13 of the 19 countries. Spending more than 30 minutes would categorize these women as not having “basic drinking water” as defined by JMP [29]. Previous studies have also reported a round-trip time greater than 30 minutes among women that water-fetch, though their finding was not limited to only mothers [8,40]. Studies have also suggested that the total time spent in water-fetching might be higher depending on the number of trips required per day and persons involved in household water-fetching [40,41].

The WHO recommends having accessible, on premise water, in part to reduce water collection time [30]. Our results do not change these policy recommendations. We found a high prevalence of water-fetching in the countries under study. Even though our research did not find an association between water-fetching and EBF, other research has reported on the numerous detriments of water fetching. Water fetching has been found to lead to psychological and emotional distress as well as musculoskeletal injury/pain [41,42]. A reduction in time spent water-fetching has also been found to be associated with reduced diarrhea prevalence, improved anthropometric indicators of child nutritional status, and a reduction in under-five mortality [11]. Women have traditionally carried much of the burden of water-fetching, [9] and this was evident in our study where in each of the 19 countries, women spent more than 20 minutes on average water-fetching, and in the most extreme country up to 115 minutes fetching water.

Our findings of no difference in EBF prevalence between mothers who fetched water and those who did not fetch water may be due to several reasons. Many mothers may value and prioritize EBF regardless of the time constraints imposed by water-fetching. Mothers in Africa often carry infants on their back during water-fetching [43], and may exclusively breastfeed their child on their trip when the child is hungry regardless of timing. It’s also possible that children are exclusively breastfed, but just not as frequently or for the same duration during times when mothers are fetching water.

While we hypothesized that mothers who perceived their water source as being clean might supplement their child’s feedings with water at an earlier age, we found that access to an improved water source was not associated with a mother’s practice of EBF. Our hypothesis was based on previous studies that reported beliefs that infants can be given water if the water is thought to be clean [16]. Our study is limited in that it measured whether the household had an improved water source, which is an imperfect indicator for whether the household had a microbiologically safe water source [20], but did not measure mothers’ perceptions of the water cleanliness.

Our study findings assessing the relationship between water, EBF and diarrhea align well with published literature. We found that EBF was associated with reduced prevalence of diarrhea in children, and this observation is consistent with previous studies on EBF and diarrhea [44-46]. We also found that access to an improved water source was not associated with diarrhea prevalence. Recent systematic reviews and rigorous trials assessing the effects of water interventions on diarrhea have been mixed [47-49].

Our study had several strengths and limitations. Strengths include that our study draws from many countries from Africa, and therefore our findings should be generalizable to the countries in our study and potentially the regions beyond our study countries. The data collection in this study was also designed to be standardized, rigorous and internationally comparable. A major limitation is that the findings cannot be interpreted causally, as this was a cross-sectional study. Another limitation is that EBF status was assessed at a single point in time using the 24-hour recall, which is known to overestimate the true prevalence of EBF practice because some infants who are given other liquids/foods irregularly may not have received them on a daily basis before the survey [50]. Although this might be a limitation in our study, the estimation of EBF status in our study is standard practice, and is consistent with international guidelines [28]. The outcomes of interest were self-reported, and thus may be subject to recall bias. However, we have no reason to expect recall to be different between mothers who water-fetched vs those who did not. Another limitation was that we could not control for some potential confounders such as previous history of EBF, knowledge on the benefits of EBF, household water sharing and seasonality (ie, wet or dry season), as these variables are not available in the MICS data. We also were unable to control for the complexity of which persons were involved in household water-fetching, for example, how many people/which persons were involved in household water-fetching. More than one person is usually involved in household water-fetching in Africa [8,40].

## CONCLUSION

This is the first study to evaluate the role of water access and household water-fetching on exclusive breastfeeding practice by mothers. We found that access to an improved water source and time spent by mothers fetching water were not associated with a mother’s practice of EBF. The study draws on data from 19 countries throughout Africa, and the findings were generally consistent across countries. At a policy level, our findings lend support to the importance of Sustainable Development Goal (SDG) target 6.1, which promotes universal access to drinking water, and the WHO call to increase the prevalence of EBF globally [34,51]. While our study was cross-sectional, future research using more rigorous designs may still be merited to understand if similar results would persist. Future research with a qualitative approach may be needed to elucidate why water access was not associated with EBF. Studies might also examine whether time spent water-fetching is associated with frequency of breastfeeding.

## Additional material

Online Supplementary Document
