# Supplementary material for: The interrelationship between water access, exclusive breastfeeding and diarrhea in children: a cross-sectional assessment across 19 African countries
Source: J Glob Health. 2021 Mar 27;11:04001. doi: 10.7189/jogh-11-04001 (PMC8005312; doi:10.7189/jogh-11-04001)
Supplement: Online Supplementary Document [file jogh-11-04001-s001.pdf]

Supplementary Files.

Figure S1. The prevalence of exclusive breastfeeding (EBF) among mothers with children aged 0-1, 2-3 and 4-5 months in 19 African countries.

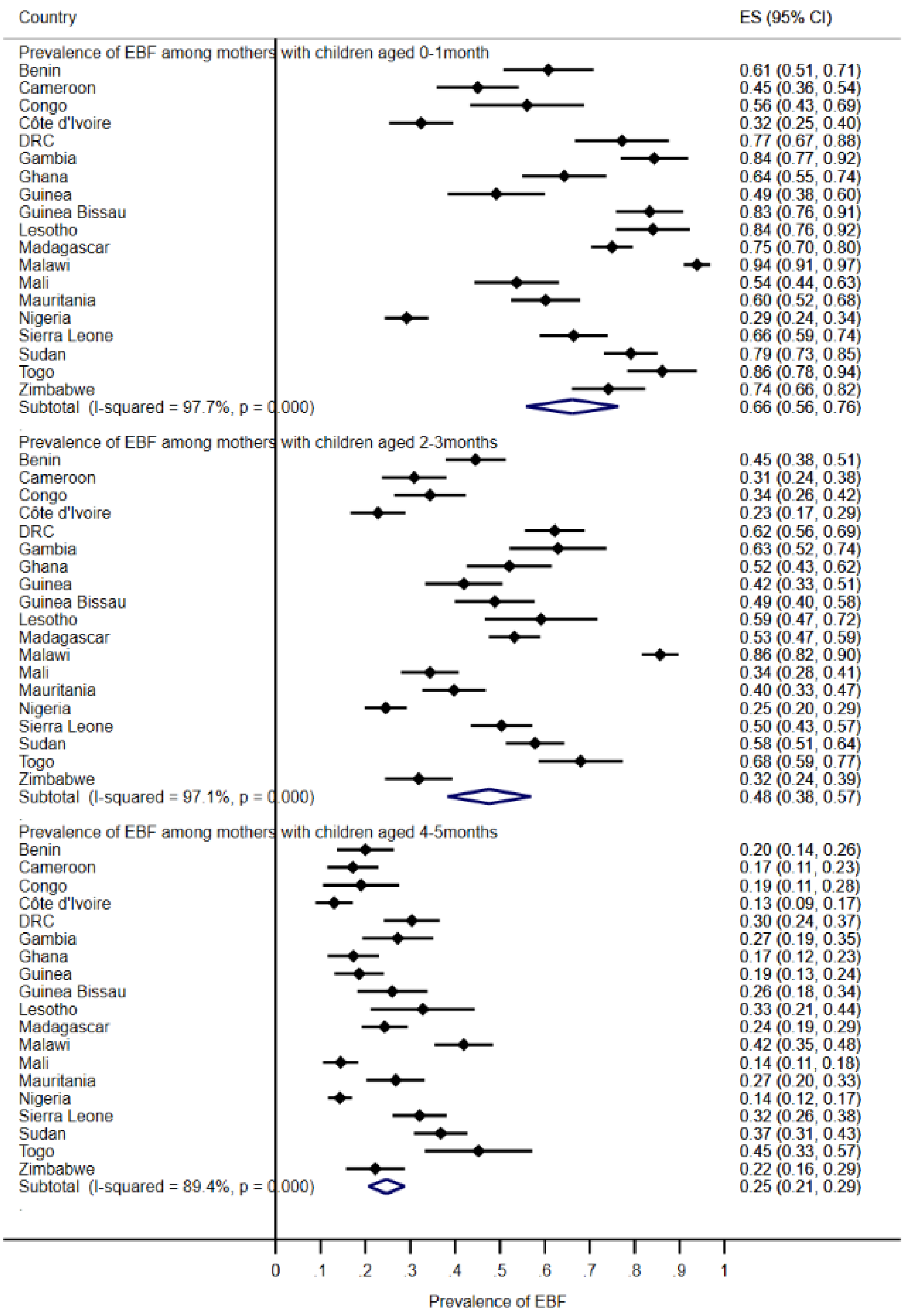

ES: Estimate of prevalence

Figure S2. The association between times spent by mothers fetching water and exclusive breastfeeding (EBF) practice among mothers in 19 African countries.

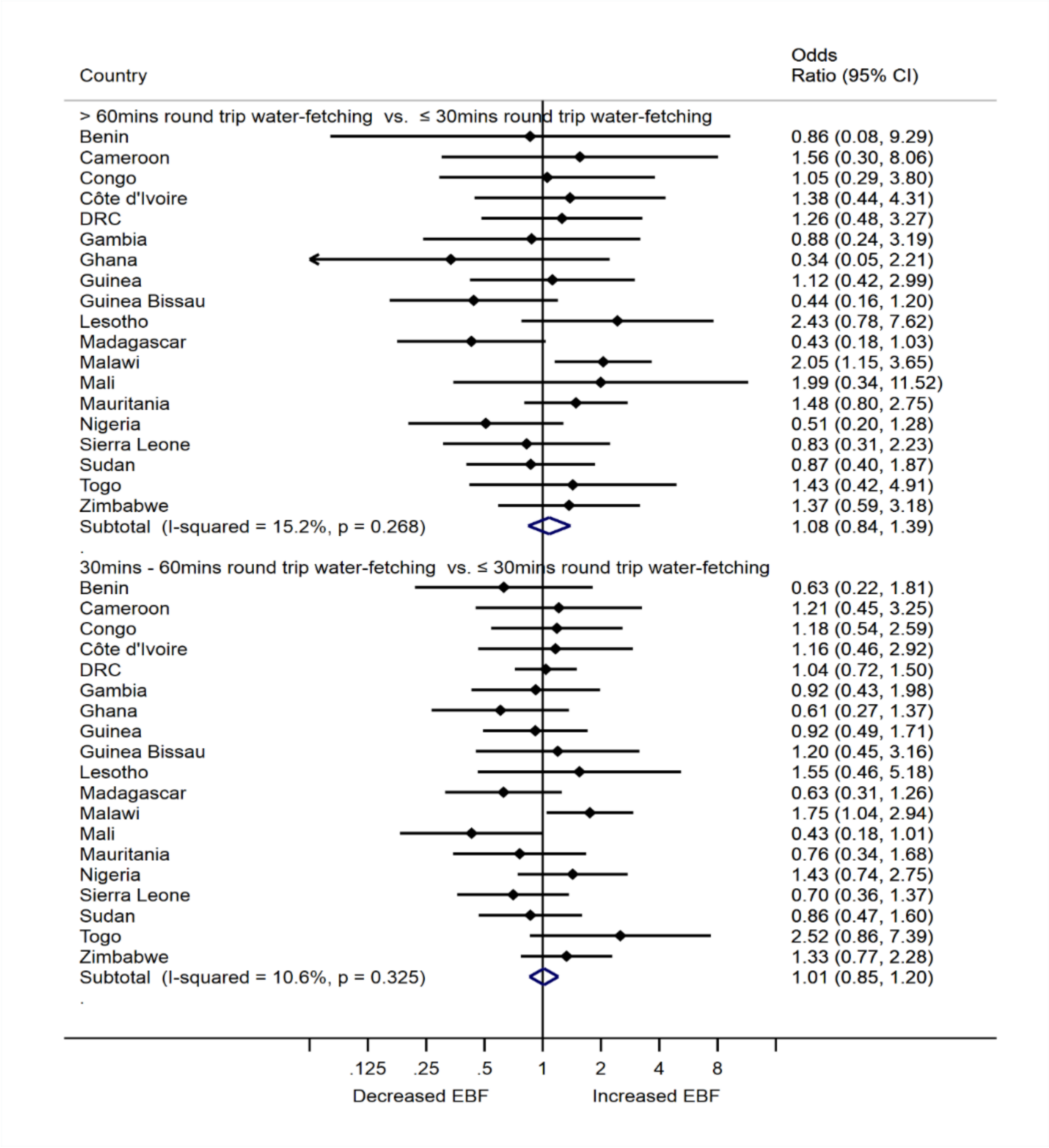

Figure S3. The association between times spent by mothers fetching water and exclusive breastfeeding (EBF) among mothers with children aged 0-1, 2-3 and 4-5 months in 19 African countries.

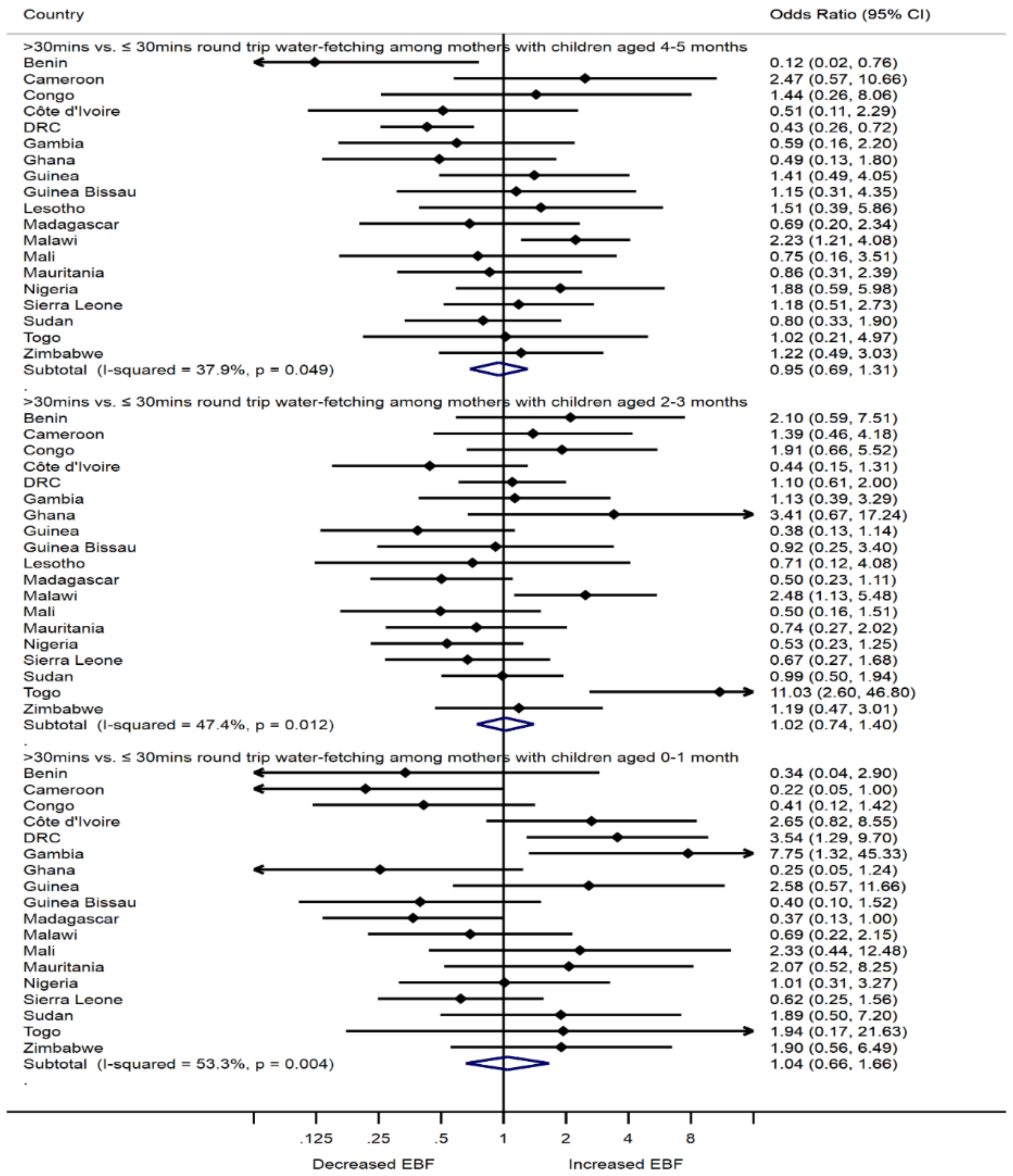

Lesotho was excluded among mothers with children aged 0-1month because of low numbers in the stratum
